# Analysis of risk factors and prognostic prediction in advanced colorectal cancer undergoing immunotherapy combined with targeted therapy

**DOI:** 10.3389/fmed.2025.1640469

**Published:** 2025-10-15

**Authors:** Yuan Yuan, Ya-Fang Chen, Xiao-Mei Liu, Ying Hu, Shuang Hao, Xin-Yan Dai

**Affiliations:** Department of Gastrointestinal Surgery, First Affiliated Hospital of Military Medical University, Chongqing, China

**Keywords:** advanced colorectal cancer, immunotherapy, targeted therapy, proficient mismatch repair, prognostic prediction, nomogram

## Abstract

**Background:**

The prognostic implications of systemic inflammatory markers in mismatch repair-proficient (pMMR) advanced colorectal cancer (CRC) treated with immunotherapy combined with targeted therapy remain unclear. This study aimed to identify key clinical and inflammatory markers predictive of overall survival (OS) and progression-free survival (PFS), and to construct a nomogram for individualized outcome prediction.

**Methods:**

This retrospective study included 216 pMMR advanced CRC patients treated with camrelizumab plus bevacizumab between January 2020 and December 2022. Baseline clinical variables and inflammatory indices, including neutrophil-to-lymphocyte ratio (NLR), cancer-inflammation prognostic index (CIPI), and systemic immune-inflammation index (SII), were analyzed. Patients were randomly assigned to a training set (*n* = 139) or a validation set (*n* = 77). Independent prognostic factors for OS and PFS were identified via multivariable Cox regression. A nomogram was constructed and internally validated using bootstrap resampling (1,000 iterations).

**Results:**

Elevated body mass index (≥25 kg/m^2^) was independently associated with improved OS (hazard ratio [HR] = 0.430; 95% CI: 0.185–0.980; *p* = 0.047), while elevated CIPI (>828.8) and carcinoembryonic antigen (>5 ng/mL) were associated with poorer OS (HR = 1.810, *p* = 0.045; HR = 2.440, *p* = 0.025, respectively). For PFS, SII ≥ 663.9 predicted worse outcomes (HR = 2.720; 95% CI: 1.200–6.200; *p* = 0.016). The nomograms demonstrated moderate discrimination with optimism-adjusted C-indices of 0.610 (PFS) and 0.650 (OS), and calibration curves showed good agreement. Kaplan–Meier analysis confirmed significantly poorer OS and PFS in high-risk groups defined by nomogram scores (*p* < 0.001 for both).

**Conclusion:**

This study highlights the prognostic significance of both clinical and inflammatory markers in pMMR advanced colorectal cancer undergoing immunotherapy combined with targeted therapy. The developed nomogram facilitates individualized survival prediction, offering clinicians a practical tool to tailor treatment and follow-up strategies for improved patient management.

## 1 Introduction

Colorectal cancer (CRC) is a common malignancy of the gastrointestinal tract and ranks as the second leading cause of cancer-related mortality worldwide. According to global cancer statistics, CRC accounts for approximately 10% of all cancer diagnoses and 9% of cancer-related deaths, with its incidence rising in both developed and developing regions. The increasing trends in CRC incidence and mortality reflect a broader global health burden. Early detection and timely therapeutic intervention are essential for improving clinical outcomes, as patients with advanced-stage CRC often experience poor survival due to distant metastasis, therapeutic resistance, and limited treatment options. The majority of patients with metastatic CRC (mCRC), particularly those harboring microsatellite-stable (MSS) or mismatch repair-proficient (pMMR) tumors, present considerable therapeutic challenges (1–3). Approximately 95% of mCRC cases are classified as pMMR, a subtype characterized by a lower response rate to immune checkpoint inhibitors compared with microsatellite instability-high (MSI-H) tumors. While MSI-H tumors typically exhibit high tumor mutational burden and neoantigen load, facilitating immune activation, pMMR tumors demonstrate lower genomic instability and an immunosuppressive tumor microenvironment, which collectively contribute to reduced efficacy of immunotherapy (4, 5).

In recent years, the combination of immunotherapy and targeted therapy has emerged as a promising treatment strategy for mCRC, particularly in pMMR tumors. Targeted agents, including epidermal growth factor receptor (EGFR) inhibitors such as cetuximab and panitumumab and vascular endothelial growth factor (VEGF) inhibitors such as bevacizumab, provide clinical benefits by disrupting critical oncogenic pathways involved in tumor progression and metastasis (6, 7). When used in conjunction with immune checkpoint inhibitors, such as programmed cell death protein 1 (PD-1) or programmed death-ligand 1 (PD-L1) inhibitors, this dual approach aims to enhance antitumor immune responses and reduce therapeutic resistance. Despite the theoretical synergy, clinical outcomes among patients with pMMR mCRC remain variable, with many individuals failing to achieve sustained responses. Identifying reliable prognostic and predictive factors is therefore essential to guide personalized treatment decisions and improve survival outcomes. While several clinical, molecular, and immunological parameters, including tumor burden, gene mutations (such as KRAS, NRAS, and BRAF), and features of the immune microenvironment, have been associated with response to therapy, no validated prognostic model has yet been established for this patient subgroup (8, 9).

This study aims to identify prognostic risk factors in patients with advanced pMMR mCRC receiving combined immunotherapy and targeted therapy. Furthermore, we seek to construct a nomogram integrating key clinical and molecular variables to enhance the accuracy of outcome prediction. This prognostic model may serve as a clinical decision-support tool to facilitate individualized treatment planning, improve survival outcomes, and reduce treatment-related toxicity.

## 2 Methods

### 2.1 Study design

**Figure 1:**
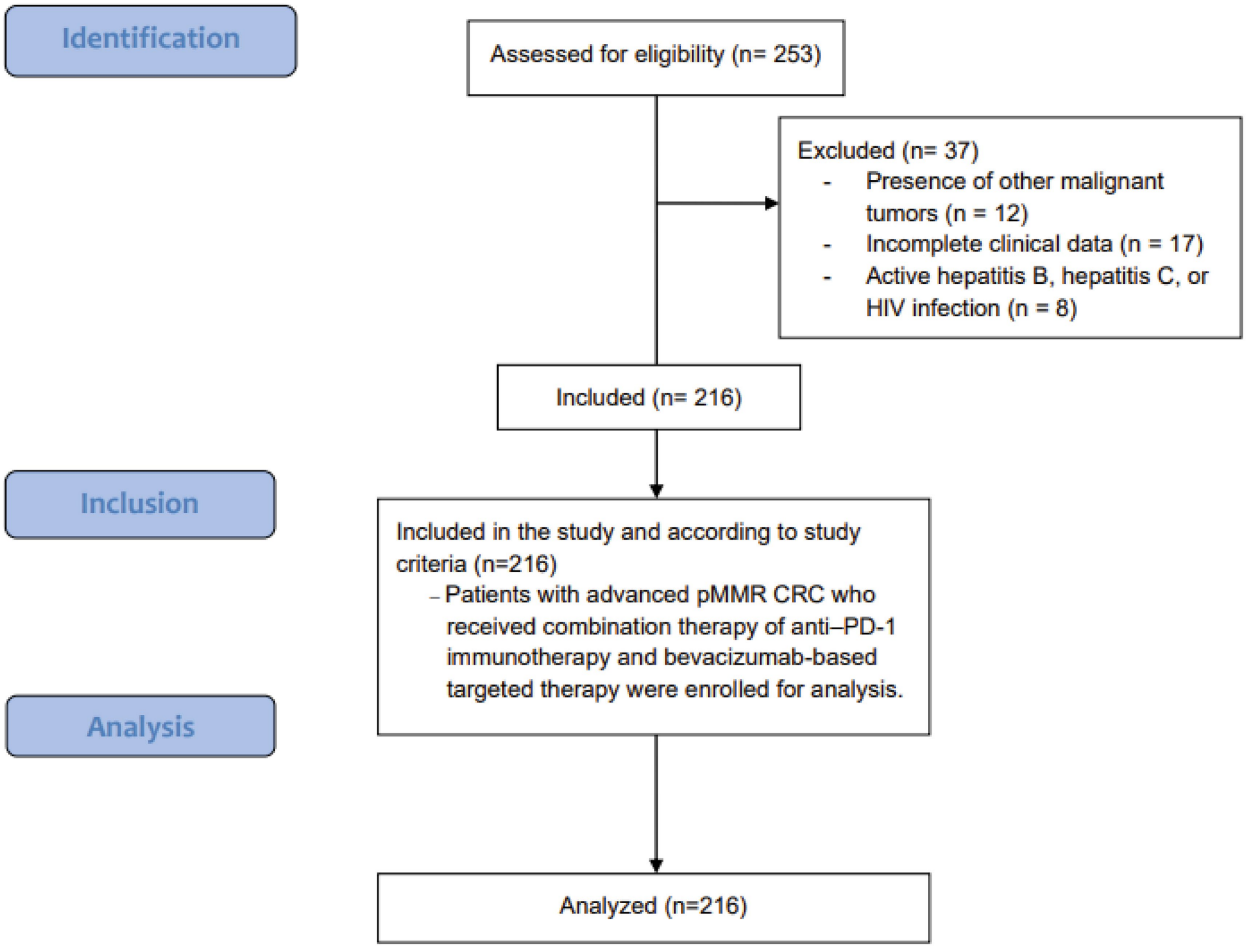
Flowchart of patient selection.

A retrospective evaluation was conducted at our institution to identify risk factors and prognostic determinants among patients with advanced CRC undergoing immunotherapy combined with targeted therapy. The study period spanned from January 2020 through December 2022. Patients were included if they met the following criteria: (1) age ≥18 years at diagnosis; (2) histopathologically confirmed CRC with clinical stage IV disease; (3) confirmed pMMR status by immunohistochemical analysis of mismatch repair proteins; (4) evidence of distant metastasis following failure of conventional treatment, subsequently receiving immunotherapy combined with targeted agents, primarily anti–PD-1 therapy plus tyrosine kinase inhibitors (TKIs) and/or VEGF monoclonal antibodies; and (5) no prior history of immunotherapy. Exclusion criteria encompassed: (1) concurrent presence of other malignant tumors; (2) incomplete clinical data; and (3) active hepatitis B, active hepatitis C, or HIV infection. A total of 216 patients were included in this analysis (Figure 1). All research methods, objectives, and protocols were formulated in accordance with the STROBE (Strengthening the Reporting of Observational Studies in Epidemiology) guidelines (10). Informed consent was obtained from all subjects and/or their legal guardian(s). The study’s methodology, intent, and protocols were reviewed and approved by the hospital’s ethics committee. All procedures adhered to applicable guidelines and the Declaration of Helsinki. Data was kept confidential, with all personal identifiers removed before analysis to protect participant privacy.

### 2.2 Treatment regimens

All patients received a uniform treatment strategy consisting of immune checkpoint inhibition in combination with targeted therapy. Immunotherapy was administered using the anti–PD-1 monoclonal antibody camrelizumab, given at standard dosing intervals. Targeted therapy included the anti–VEGF monoclonal antibody bevacizumab. The selection of these agents was based on institutional protocol and clinical consensus. Importantly, all patients received combination therapy of camrelizumab plus bevacizumab, ensuring consistency of therapeutic modality across the cohort. No patients received EGFR inhibitors or prior immunotherapy.

### 2.3 Data collection and outcome measures

Baseline clinical data were collected for all patients. This included demographic variables such as sex and age, physical measurements including height and weight, tumor location, complete blood count (CBC) parameters, and tumor marker levels. Key indices were derived from these data to assess inflammatory and prognostic markers.

The body mass index (BMI) was calculated as weight (kg) divided by height squared (m^2^). The NLR was computed by dividing the neutrophil count by the lymphocyte count. The cancer-inflammation prognostic index (CIPI) was determined using the formula: carcinoembryonic antigen (CEA) level × neutrophil count/lymphocyte count. The systemic immune-inflammation index (SII) was calculated as platelet count × neutrophil count/lymphocyte count. These indices provide valuable insights into the systemic inflammatory and immune response of the patients.

To evaluate the prognostic value of NLR, CIPI, and SII, their optimal cutoff values were identified using X-tile software (version 3.6.1, Yale University), with overall survival (OS) as the primary endpoint. The resulting cutoff thresholds were 3.05 for NLR, 828.8 for CIPI, and 663.9 for SII. Patients were categorized into high- and low-risk groups based on these thresholds. BMI was classified according to the World Health Organization (WHO) criteria as ≥25 or <25 kg/m^2^ (11). CEA levels were divided into two groups: ≤5 ng/mL or >5 ng/mL, based on standard laboratory reference ranges. These classifications enabled stratification of patients for further analysis (12, 13).

### 2.4 Study outcomes and follow-up

Follow-up was conducted through telephone and outpatient visits, with a primary focus on assessing patient disease progression and survival status. Follow-up visits were scheduled every 3 months, with the final follow-up date set for September 2024. Progression-free survival (PFS) was defined as the time interval from the initiation of immunotherapy to disease progression, death, loss to follow-up, or the end of the study period. OS was defined as the time from the start of immunotherapy to death, loss to follow-up, or the end of the study period.

### 2.5 Statistical analysis

Statistical analyses were performed using SPSS version 27.0 and R version 4.1.3 software. Patients were randomly allocated to a training set and a validation set. The training set was utilized for constructing the predictive model, while the validation set served to evaluate its performance. Categorical variables were presented as frequencies (n) and percentages (%). OS and PFS were estimated using the Kaplan–Meier method, and survival curves were compared between groups using the Log-rank test. For Cox regression analyses, the proportional hazards (PH) assumption was formally tested using Schoenfeld residuals (global and covariate-specific tests), and scaled Schoenfeld residual plots were examined to exclude time-dependent effects; no significant violations were observed. To identify independent prognostic factors, a multivariate Cox proportional hazards regression model was employed, calculating hazard ratios (HR) and 95% confidence intervals (95% CI) for each variable. Based on the significant prognostic factors identified, a nomogram was developed to predict individual patient outcomes. The discriminative ability of the model was evaluated using receiver operating characteristic (ROC) curves, with the area under the curve (AUC) serving as a measure of model performance. An AUC closer to 1 indicated high predictive accuracy, whereas an AUC near 0.5 suggested poor discrimination. Additionally, the model’s consistency was assessed using the concordance index (C-index) and calibration curves to compare predicted versus observed outcomes. Internal validation was conducted using bootstrap resampling with 1,000 iterations. In each iteration, the model was refitted on a bootstrap sample and tested on the original dataset to estimate optimism. Optimism-corrected C-indices, time-dependent AUCs, and calibration parameters (intercept and slope) were derived to quantify model robustness and reduce overfitting. Patients were assigned nomogram scores, which were then used to stratify them into high-risk and low-risk groups using X-tile software. Survival differences between these risk groups were analyzed with the Log-rank test. All statistical tests were two-sided, with a significance threshold set at *p* < 0.05.

## 3 Results

### 3.1 Patient demographics and clinical outcomes

This study included a total of 216 eligible patients with pMMR advanced CRC who underwent immunotherapy combined with targeted therapy. The age of participants ranged from 25 to 83 years, with a median age of 57 years. The cohort consisted of 120 males (55.6%) and 96 females (44.4%). Over a median follow-up period of 26 months, 198 patients (91.7%) experienced disease progression, and 185 patients (85.6%) died of the disease. The OS rates at 3, 6, and 12 months were 91.2% (197/216), 63.0% (136/216), and 30.6% (66/216), respectively. The median OS was recorded at 9 months, while the median PFS was 5 months. For analytical purposes, patients were randomly divided into a training cohort (*n* = 139) and a validation cohort (*n* = 77). Baseline clinical characteristics before the initiation of immunotherapy combined with targeted therapy were compared between the two groups. The comparison revealed no statistically significant differences in variables such as sex, age, drinking and smoking status, primary tumor focus, BMI, CEA levels, and inflammatory prognostic indices (all *p* > 0.05), as detailed in Table 1.

**Table 1:** Comparison of baseline characteristics between training cohort and validation cohort.

Variables	Training cohort (*n* = 139)	Validation cohort (*n* = 77)
Sex		
Male	80 (57.6%)	40 (51.9%)
Female	59 (42.4%)	37 (48.1%)
Age (years)		
<60	81 (58.3%)	49 (63.6%)
≥60	58 (41.7%)	28 (36.4%)
Drinking		
No	113 (81.3%)	72 (93.5%)
Yes	26 (18.7%)	5 (6.5%)
Smoking		
No	109 (78.4%)	72 (93.5%)
Yes	30 (21.6%)	5 (6.5%)
Primary focus		
Right half	42 (30.2%)	23 (29.9%)
Left half	97 (69.8%)	54 (70.1%)
BMI (kg/m^2^)		
<25	117 (84.2%)	62 (80.5%)
≥25	22 (15.8%)	15 (19.5%)
CEA (ng/mL)		
≤5	26 (18.7%)	18 (23.4%)
>5	113 (81.3%)	59 (76.6%)
SII		
<663.9	88 (63.3%)	54 (70.1%)
≥663.9	51 (36.7%)	23 (29.9%)
CIPI		
<828.8	95 (68.3%)	62 (80.5%)
≥828.8	44 (31.7%)	15 (19.5%)
NLR		
<3.05	80 (57.6%)	49 (63.6%)
≥3.05	59 (42.4%)	28 (36.4%)

CEA, carcinoembryonic antigen; BMI, body mass index; CIPI, cancer-inflammation prognostic index; NLR, neutrophil-to-lymphocyte ratio; SII: systemic immune-inflammation index.

### 3.2 Univariable analysis of OS and PFS in patients with pMMR advanced CRC receiving immunotherapy combined with targeted therapy

In the univariable analysis, several clinical and biochemical factors were significantly associated with OS and PFS in patients with pMMR advanced CRC undergoing immunotherapy combined with targeted therapy. A higher BMI (≥25 kg/m^2^) was linked to improved OS, with a median OS of 14.2 months compared to 7.6 months for those with BMI < 25 kg/m^2^ (*p* = 0.010). Elevated SII (≥663.9) and CIPI (≥828.8) were both associated with poorer OS, with median survival times of 5.1 and 4.6 months, respectively, versus longer survival in patients with lower SII and CIPI values (*p* = 0.001 and *p* = 0.002). For PFS, elevated CEA (>5 ng/mL), SII, and CIPI were significant predictors of shorter progression-free intervals, with median PFS of 3.6 months in the high-CEA group versus 4.0 months in the low-CEA group (*p* = 0.018), and 2.6 months versus 4.1 months for high versus low SII (*p* = 0.002) and CIPI (*p* = 0.014). Additionally, an increased NLR (≥3.05) was associated with worse OS and PFS (*p* = 0.002 and *p* = 0.040, respectively). In contrast, variables such as sex, age, alcohol consumption, smoking status, and primary tumor location were not significantly correlated with survival outcomes (Table 2).

**Table 2:** Univariable analysis of OS and PFS in patients with pMMR advanced CRC receiving immunotherapy combined with targeted therapy.

Variables	Median OS (95% CI), months	*p*-value	Median PFS (95% CI), months	*p*-value
BMI (kg/m^2^)		0.010		0.225
<25	7.6 (5.1–10.0)		4.0 (3.3–4.7)	
≥25	14.2 (11.3–16.8)		3.4 (1.8–5.3)	
CEA (ng/mL)		0.085		0.018
≤5	12.4 (7.2–17.8)		4.0 (0.0–11.6)	
>5	8.2 (5.3–10.7)		3.6 (2.8–4.2)	
SII		0.001		0.002
<663.9	10.7 (9.2–12.9)		4.1 (3.2–4.8)	
≥663.9	5.1 (1.6–8.5)		2.6 (1.4–3.6)	
CIPI		0.002		0.014
<828.8	10.4 (8.4–12.6)		4.0 (3.2–4.8)	
≥828.8	4.6 (3.2–6.0)		2.6 (1.5–3.5)	
NLR		0.002		0.040
<3.05	10.9 (9.2–12.8)		4.0 (3.6–4.4)	
≥3.05	5.8 (2.7–9.4)		2.6 (1.3–3.7)	
Sex		0.214		0.745
Male	8.4 (6.1–10.9)		4.2 (3.0–5.1)	
Female	7.8 (5.1–10.5)		3.6 (2.7–4.4)	
Age (years)		0.231		0.814
<60	9.4 (7.1–11.6)		4.1 (3.3–4.9)	
≥60	6.8 (3.6–10.5)		3.6 (2.4–4.6)	
Drinking		0.642		0.899
No	7.9 (5.3–10.6)		4.0 (3.3–4.7)	
Yes	12.3 (7.0–17.5)		4.1 (2.3–5.8)	
Smoking		0.530		0.675
No	8.3 (6.7–10.1)		4.0 (3.4–4.6)	
Yes	7.7 (3.7–12.2)		4.1 (2.1–5.9)	
Primary focus		0.657		0.623
Right half	7.9 (6.8–9.1)		3.1 (1.5–4.4)	
Left half	8.4 (5.7–11.4)		4.1 (3.1–4.9)	

OS, overall survival; PFS, progression-free survival; BMI, body mass index; CEA, carcinoembryonic antigen; CIPI, cancer-inflammation prognostic index; SII, systemic immune-inflammation index; NLR, neutrophil-to-lymphocyte ratio.

### 3.3 Multivariable analysis of OS and PFS in patients with pMMR advanced CRC receiving immunotherapy combined with targeted therapy

In the multivariable analysis, several factors were evaluated for their impact on OS and PFS in patients with pMMR advanced CRC undergoing immunotherapy combined with targeted therapy. A BMI of ≥25 kg/m^2^ was significantly associated with improved OS (HR = 0.430, 95% CI: 0.185–0.980, *p* = 0.047). Elevated CIPI (>828.8) was linked to poorer OS (HR = 1.810, 95% CI: 1.002–3.260, *p* = 0.045). Higher CEA levels (>5 ng/mL) were also associated with reduced OS (HR = 2.440, 95% CI: 1.120–5.350, *p* = 0.025). Regarding PFS, a higher SII (≥663.9) was significantly associated with shorter PFS (HR = 2.720, 95% CI: 1.200–6.200, *p* = 0.016). Neither BMI nor CIPI showed a significant association with PFS (*p* = 0.225 and *p* = 0.380, respectively). Additionally, the NLR (≥3.05) did not significantly impact OS or PFS (HR = 1.220, 95% CI: 0.540–2.750, *p* = 0.612 for OS and HR = 0.640, 95% CI: 0.290–1.430, *p* = 0.285 for PFS) (Table 3).

**Table 3:** Multivariable analysis of OS and PFS in patients with pMMR advanced CRC receiving immunotherapy combined with targeted therapy.

Variables	HR (95% CI) OS	*p*-value	HR (95% CI) PFS	*p*-value
BMI (≥25 kg/m^2^ vs. < 25 kg/m^2^)	0.430 (0.185–0.980)	0.047	-	-
CIPI (>828.8 vs. < 828.8)	1.810 (1.002–3.260)	0.045	1.290 (0.720–2.330)	0.380
CEA (>5 ng/mL vs. ≤ 5 ng/mL)	2.440 (1.120–5.350)	0.025	-	-
SII (≥663.9 vs. < 663.9)	1.720 (0.770–3.800)	0.182	2.720 (1.200–6.200)	0.016
NLR (≥3.05 vs. < 3.05)	1.220 (0.540–2.750)	0.612	0.640 (0.290–1.430)	0.285

OS, overall survival; PFS, progression-free survival; BMI, body mass index; CEA, carcinoembryonic antigen; CIPI, cancer-inflammation prognostic index; SII, systemic immune-inflammation index; NLR, neutrophil-to-lymphocyte ratio.

### 3.4 Construction of the nomogram model for predicting outcomes in patients with pMMR advanced CRC

**Figure 2:**
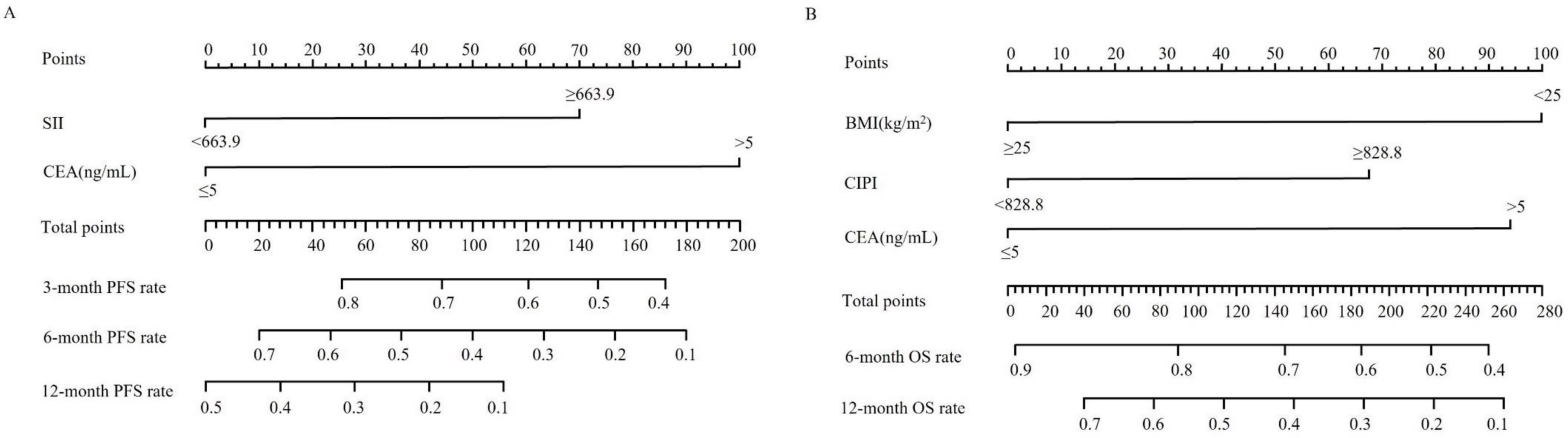
Nomograms for individualized prediction of survival outcomes in patients with advanced CRC receiving immunotherapy combined with targeted therapy. **(A)** Nomogram for estimating 3-, 6-, and 12-month progression-free survival (PFS) based on systemic immune-inflammation index (SII) and carcinoembryonic antigen (CEA). **(B)** Nomogram for estimating 6- and 12-month overall survival (OS) based on body mass index (BMI), cancer-inflammation prognostic index (CIPI), and CEA. To use the nomogram, locate the patient’s value for each predictor variable, draw a vertical line upward to determine the corresponding number of points, sum the total points, and project downward to estimate the probability of survival at each time point.

Based on the independent prognostic factors identified through multivariable Cox regression analysis, a nomogram model was developed to predict survival outcomes in patients with pMMR advanced CRC receiving immunotherapy combined with targeted therapy. Each significant variable contributes a specific point value, and the sum of these point values constitutes a total score. By aligning this total score with the scales presented at the bottom of the nomogram, individualized probabilities of PFS (Figure 2A) and OS (Figure 2B) can be estimated. Higher total scores correlate with a greater likelihood of disease progression and shorter survival, while lower total scores suggest more favorable outcomes.

### 3.5 Discriminative capacity of the nomogram models for PFS and OS

**Figure 3:**
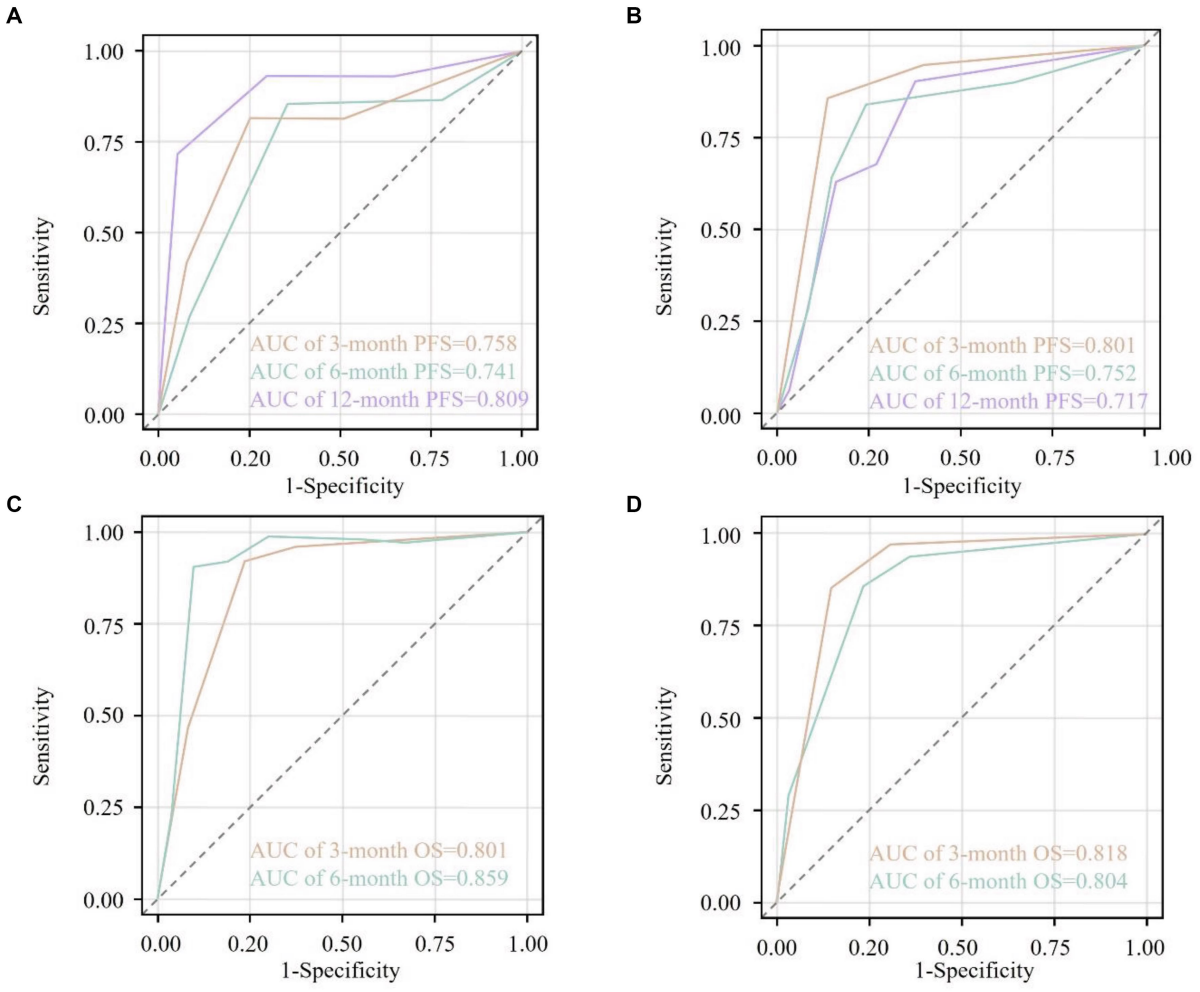
Receiver operating characteristic (ROC) curves for the nomogram-predicted survival outcomes. **(A)** ROC curves at 3 months (orange), 6 months (cyan), and 12 months (purple) for progression-free survival (PFS) in the training cohort. **(B)** ROC curves at 3 months (orange), 6 months (cyan), and 12 months (purple) for PFS in the validation cohort. **(C)** ROC curves at 3 months (orange) and 6 months (cyan) for overall survival (OS) in the training cohort. **(D)** ROC curves at 3 months (orange) and 6 months (cyan) for OS in the validation cohort. The area under the curve (AUC) values are annotated within each plot, indicating the discriminative ability of the nomograms at different time points. The diagonal dashed line represents a non-informative model (AUC = 0.5). AUC values closer to 1.0 indicate higher predictive accuracy.

The predictive accuracy of the constructed nomograms was evaluated using the C-index and ROC curves in both the training and validation cohorts. For the PFS nomogram, the C-index was 0.615 in the training cohort and 0.598 in the validation cohort. ROC curve analysis further showed that, in the training cohort, the AUC values at 3, 6, and 12 months were 0.758, 0.741, and 0.809, respectively (Figure 3A), whereas in the validation cohort these values were 0.801, 0.752, and 0.717 (Figure 3B). These findings indicate that the model’s ability to discriminate between patients with different PFS outcomes was relatively modest. For the OS nomogram, the training cohort had a C-index of 0.658, and the validation cohort had a C-index of 0.665. In the training cohort, the AUC values at 6 and 12 months were 0.801 and 0.859, respectively (Figure 3C), while in the validation cohort they were 0.818 and 0.804 (Figure 3D). These results suggest a moderate level of discriminatory power for OS, with the nomogram exhibiting reasonable accuracy in identifying patients at higher risk of mortality.

### 3.6 Internal validation and calibration

Bootstrap resampling with 1,000 iterations confirmed the robustness of the nomogram models. For PFS, the optimism-corrected C-index was 0.610 (apparent: 0.615), and the optimism-adjusted AUCs at 3, 6, and 12 months were 0.740, 0.720, and 0.790, respectively, closely aligning with the apparent estimates. For OS, the optimism-corrected C-index was 0.650 (apparent: 0.658), with corrected AUCs of 0.800 and 0.840 at 6 and 12 months, respectively. Calibration curves demonstrated strong concordance between predicted and observed probabilities in both the training and validation cohorts. Calibration intercepts were close to zero, and slopes approximated one for both PFS and OS, indicating no systematic over- or under-estimation of risk.

### 3.7 Risk stratification based on nomogram scores

**Figure 4:**
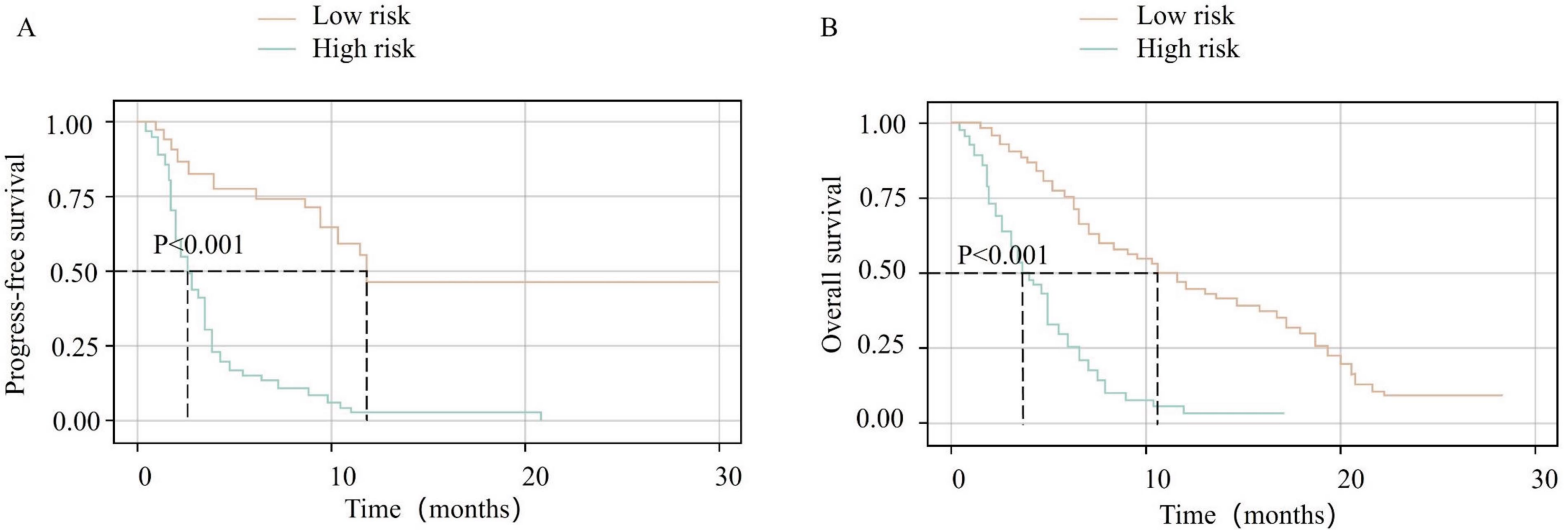
Kaplan–Meier survival curves stratified by nomogram-derived risk scores. **(A)** Progression-free survival (PFS) for high-risk (total points ≥70) vs. low-risk (total points <70) groups. **(B)** Overall survival (OS) for high-risk (total points ≥162) vs. low-risk (total points <162) groups. Risk groups were defined using optimal cutoff values determined by X-tile analysis. Survival curves are colored by risk category: low-risk group (orange line) and high-risk group (green line). Statistical differences between groups were assessed using the log-rank test (*p* < 0.001 for both PFS and OS).

Using x-tile software to analyze the nomogram-derived prediction scores for PFS and OS, optimal cutoff values were determined at 70 and 162 points, respectively. According to these thresholds, patients were categorized into high- and low-risk groups. In the PFS cohort, 178 patients had scores ≥70 (high-risk group) with a median PFS of 3 months, whereas 38 patients had scores <70 (low-risk group) with a median PFS of 12 months. The Kaplan–Meier curves indicated significantly lower PFS rates in the high-risk group compared with the low-risk group (*p* < 0.001, Figure 4A). For OS, 61 patients had scores ≥162 (high-risk group), showing a median OS of 4 months, while 155 patients with scores <162 (low-risk group) exhibited a median OS of 11 months. The high-risk group’s OS rate was significantly lower than that of the low-risk group (*p* < 0.001, Figure 4B).

## 4 Discussion

The treatment paradigm for advanced CRC has undergone substantial advancement with the introduction of immunotherapy and targeted agents; however, clinical outcomes remain highly variable. This study aimed to identify and validate key prognostic factors associated with survival and disease progression in patients with mismatch repair–proficient (pMMR) advanced CRC. Specifically, we evaluated the prognostic relevance of BMI, CEA, and inflammatory markers, including the SII, CIPI, and NLR. These biomarkers were selected for their established roles in reflecting systemic inflammation, tumor burden, and nutritional status—parameters that influence tumor biology and therapeutic response (14–16). In a study of 216 patients with pMMR advanced CRC treated with combined immunotherapy and targeted therapy, several clinical and inflammatory indicators demonstrated prognostic significance. Higher BMI, lower SII, and lower CIPI were associated with prolonged OS, whereas elevated CEA levels correlated with poorer OS. Multivariable Cox regression analysis identified BMI ≥ 25 kg/m^2^, CIPI >828.8, CEA > 5 ng/mL, and SII ≥ 663.9 as independent predictors of clinical outcomes.

First, the observation that patients with a higher BMI (≥25 kg/m^2^) exhibited improved OS contrasts with the common belief that obesity worsens oncologic outcomes. This finding may reflect better nutritional status, which could enhance treatment tolerance and immune function. Additionally, adipose tissue serves as a reservoir for immunomodulatory cytokines that may boost antitumor immune responses, especially during immunotherapy (17). While obesity is linked to chronic low-grade inflammation, which can both promote tumor growth and support antitumor immunity, this relationship is complex. Proinflammatory cytokines from adipose tissue may recruit immune cells, such as T-cells, to the tumor site, enhancing immune surveillance (18, 19). However, the BMI-tumor biology relationship is multifaceted, and further research is needed to clarify its underlying mechanisms and clinical implications for advanced CRC patients undergoing combined immunotherapy and targeted therapy (20–22). Second, elevated SII (≥663.9) and CIPI (≥828.8) were significantly associated with worse survival outcomes. These indices reflect systemic inflammatory status by integrating hematological parameters such as platelet, neutrophil, and lymphocyte counts. Persistent systemic inflammation is known to foster a pro-tumorigenic microenvironment by promoting angiogenesis, tumor proliferation, and immune evasion through various cytokines and growth factors. Accordingly, elevated SII and CIPI may indicate a more aggressive disease phenotype and a suppressed antitumor immune response, thereby reducing the efficacy of immune-based therapies (23, 24). Third, elevated CEA (>5 ng/mL) was associated with inferior survival outcomes. As a well-established tumor marker in CRC, high CEA levels often reflect greater tumor burden and metastatic potential. This finding is consistent with prior reports correlating elevated CEA with advanced disease stage and reduced therapeutic benefit, even in the setting of immunotherapy. High CEA concentrations may indicate not only an increased tumor load but also an immunosuppressive tumor microenvironment, contributing to reduced responsiveness to immune checkpoint blockade (25, 26).

Although the NLR (≥3.05) was significantly associated with outcomes in univariable analyses, it did not retain independent prognostic significance for either OS or PFS in multivariable models. This discrepancy suggests that, while NLR may reflect systemic inflammation, its prognostic value is diminished when considered alongside more comprehensive indices such as the SII and the CIPI, which integrate additional hematologic components. The overlapping biological information captured by these markers may account for the attenuated role of NLR in the final predictive model. The nomogram models constructed using these independent predictors demonstrated modest to moderate discriminative ability, as reflected by the C-index and AUC values. Specifically, the OS nomogram exhibited moderate predictive accuracy, while the PFS nomogram demonstrated relatively limited discriminative capacity. The lower performance of the PFS model may be attributed to the biologic heterogeneity of metastatic CRC, where progression can be influenced by dynamic factors such as evolving therapeutic regimens, emergence of drug resistance, and changes within the tumor microenvironment over time (27, 28). In contrast, OS may be more consistently influenced by baseline clinical and inflammatory characteristics, thereby enhancing the model’s predictive strength. Importantly, the risk stratification derived from the nomogram-based cutoff values highlights the clinical applicability of these models. By categorizing patients into high- and low-risk groups for PFS and OS, clinicians may be better equipped to individualize treatment plans, optimize follow-up intensity, and identify candidates for alternative therapeutic strategies aimed at improving long-term outcomes.

While the C-index values for PFS (ranging from 0.598 in the validation cohort to 0.615 in the training cohort) and OS (0.650 optimism-corrected and 0.658 apparent) suggest only modest discriminatory power, the nomogram remains clinically useful in risk stratification for patients with pMMR advanced CRC undergoing immunotherapy combined with targeted therapy. Even with limited accuracy, this model can help identify high-risk patients who may benefit from more aggressive monitoring or alternative treatment strategies, particularly in the complex and heterogeneous landscape of mCRC. The relatively higher AUC values at specific time points, especially for OS, highlight the model’s utility in distinguishing short-term survival differences. To improve predictive accuracy, future studies should incorporate molecular and imaging biomarkers, such as genetic mutations (e.g., KRAS, NRAS, BRAF), microsatellite instability (MSI) status, and radiomics-derived imaging features. These biomarkers provide deeper insights into tumor biology, resistance mechanisms, and immune responses, thus enhancing the model’s capacity for more personalized treatment strategies. Integrating such parameters with clinical and inflammatory indices will likely refine the model, further improving its clinical applicability in advanced CRC.

The inflammatory indices identified in this study, including the SII, CIPI, and NLR, provide valuable insights into the immune and inflammatory landscape of advanced CRC patients undergoing immunotherapy combined with targeted therapy. These indices significantly correlated with PFS and OS, highlighting their prognostic value. However, to enhance the predictive accuracy and clinical applicability of this model, emerging biomarkers such as ctDNA, mutational load, and MSI status should be incorporated. For instance, ctDNA can capture minimal residual disease and dynamic changes in tumor burden, providing real-time assessment of treatment response (29). Similarly, mutational load and MSI status are critical in evaluating tumor immunogenicity and response to immunotherapy. By combining these molecular markers with inflammatory indices, clinicians could achieve a more robust and comprehensive understanding of tumor biology, ultimately refining patient risk stratification and treatment personalization (30). The risk stratification model presented in this study provides an essential framework for tailoring treatment strategies based on individual patient risk profiles. For high-risk patients, who are predicted to have poorer survival outcomes based on their nomogram scores, clinicians may consider more aggressive monitoring and potential adjustments in therapeutic regimens. For example, these patients could be prioritized for second-line or experimental therapies, or enrolled in clinical trials, aiming to improve survival (31). Additionally, intensified surveillance for early detection of disease progression or recurrence may be beneficial. On the other hand, low-risk patients with favorable prognostic factors may benefit from standard treatment protocols with less frequent monitoring (32). These patients could experience fewer toxicities, which could improve their quality of life. By implementing these stratified approaches, healthcare providers can optimize resource allocation and treatment intensity, leading to personalized care that enhances both the clinical and emotional outcomes for CRC patients.

This study has several limitations. First, its retrospective, single-center design introduces potential selection bias and limits the generalizability of the findings, despite the use of strict inclusion criteria and standardized treatment protocols. Second, although the sample size was adequate for exploratory analysis, it may have limited statistical power, which could affect the identification of additional prognostic factors. Third, missing data was minimal and handled via complete-case analysis, but this approach may still introduce bias and affect the robustness of the results. Finally, the prognostic nomograms were internally validated within the same cohort without external validation, which restricts their applicability to broader, more diverse populations. Future studies should focus on conducting prospective, multicenter investigations with larger and more diverse cohorts to externally validate and refine the proposed models. Additionally, longer follow-up periods and the inclusion of novel biomarkers, such as molecular signatures or imaging parameters, could further improve predictive accuracy and offer a deeper understanding of the biological mechanisms that drive tumor progression and therapeutic response.

## 5 Conclusion

In summary, this study underscores the prognostic significance of both clinical and inflammatory parameters, particularly BMI, CEA, SII, and CIPI, in patients with pMMR advanced CRC receiving immunotherapy combined with targeted therapy. The developed nomogram enabled individualized risk stratification and demonstrated potential to support clinical decision-making, representing a promising tool for advancing personalized treatment strategies in advanced CRC.

## Data Availability

The raw data supporting the conclusions of this article will be made available by the authors, without undue reservation.
